# The Use of Web-Based Support Groups Versus Usual Quit-Smoking Care for Men and Women Aged 21-59 Years: Protocol for a Randomized Controlled Trial

**DOI:** 10.2196/16417

**Published:** 2020-01-14

**Authors:** Cornelia Ann Pechmann, Douglas Calder, Connor Phillips, Kevin Delucchi, Judith J Prochaska

**Affiliations:** 1 The Paul Merage School of Business University of California, Irvine Irvine, CA United States; 2 School of Medicine University of California, San Francisco San Francisco, CA United States; 3 Stanford Prevention Research Center Stanford University Stanford, CA United States

**Keywords:** smoking prevention, support group, cigarettes, tobacco

## Abstract

**Background:**

Existing smoking cessation treatments are challenged by low engagement and high relapse rates, suggesting the need for more innovative, accessible, and interactive treatment strategies. Twitter is a Web-based platform that allows people to communicate with each other throughout the day using their phone.

**Objective:**

This study aims to leverage the social media platform of Twitter for fostering peer-to-peer support to decrease relapse with quitting smoking. Furthermore, the study will compare the effects of coed versus women-only groups on women’s success with quitting smoking.

**Methods:**

The study design is a Web-based, three-arm randomized controlled trial with two treatment arms (a coed or women-only Twitter support group) and a control arm. Participants are recruited online and are randomized to one of the conditions. All participants will receive 8 weeks of combination nicotine replacement therapy (patches plus their choice of gum or lozenges), serial emails with links to Smokefree.gov quit guides, and instructions to record their quit date online (and to quit smoking on that date) on a date falling within a week of initiation of the study. Participants randomized to a treatment arm are placed in a fully automated Twitter support group (coed or women-only), paired with a buddy (matched on age, gender, location, and education), and encouraged to communicate with the group and buddy via daily tweeted discussion topics and daily automated feedback texts (a positive tweet if they tweet and an encouraging tweet if they miss tweeting). Recruited online from across the continental United States, the sample consists of 215 male and 745 female current cigarette smokers wanting to quit, aged between 21 and 59 years. Self-assessed follow-up surveys are completed online at 1, 3, and 6 months after the date they selected to quit smoking, with salivary cotinine validation at 3 and 6 months. The primary outcome is sustained biochemically confirmed abstinence at the 6-month follow-up.

**Results:**

From November 2016 to September 2018, 960 participants in 36 groups were recruited for the randomized controlled trial, in addition to 20 participants in an initial pilot group. Data analysis will commence soon for the randomized controlled trial based on data from 896 of the 960 participants (93.3%), with 56 participants lost to follow-up and 8 dropouts.

**Conclusions:**

This study combines the mobile platform of Twitter with a support group for quitting smoking. Findings will inform the efficacy of virtual peer-to-peer support groups for quitting smoking and potentially elucidate gender differences in quit rates found in prior research.

**Trial Registration:**

ClinicalTrials.gov NCT02823028; https://clinicaltrials.gov/ct2/show/NCT02823028

**International Registered Report Identifier (IRRID):**

DERR1-10.2196/16417

## Introduction

### Background

State tobacco quitlines have demonstrated efficacy but remain underutilized, reaching an average of only 1% of smokers annually [1]. Dozens of randomized controlled trials have examined websites with advanced features such as QuitNet [2,3], or one-way SMS text messaging or email messaging services such as txt2stop [4,5], but these interventions are limited by information exchanges that are largely unilateral, noninteractive, and nonpeer-based. Notably, although the initial results of one-way messaging services for smoking cessation looked promising [4,6-8], a recent review found that only 3 of 15 randomized trials showed significant benefits [9].

To increase utilization of quit-smoking programs, researchers and practitioners are seeking to harness the power of social media [10]. Very popular social media sites such as Facebook and Twitter allow users to stay connected to individuals and groups in real time and to share content at virtually no cost [11]. Social media is entrenched in the United States with 73% of online adults using social media sites, 42% using multiple sites, and the majority visiting them daily [12]. Twitter has a reported 326 million active users (100 million daily) posting 500 million tweets daily, with 80% using the platform on their phone in 2018 [13]. There are already over 140 reported medical and health care uses of Twitter [14]. Relative to Facebook, Twitter has a superior application programming interface, which makes it easier to create programs for research and intervention purposes [15]. In addition, Twitter does a much better job of ensuring that group member communications stay within the group only by readily allowing for private groups to be set up that are completely and permanently isolated from friends, family, and the public.

### Objectives

Social media looks highly promising for delivering tobacco and other addiction treatment interventions as a result of its broad reach and appeal, real-time interactivity, and free cost. However, despite its apparent promise and observational evidence indicating that existing socially mediated forums may increase patient compliance [16], social media’s potential for delivering health interventions and developing new treatment approaches has not yet been fully realized. The earliest forms of social media interventions tended to be large online health forums, and these generally did not yield significant benefits [17-21], although the people who were actively engaged did often benefit [22]. Thus, the main problem with prior social media interventions seems to be that engagement was too low [23]. Our Twitter-based intervention called Tweet2Quit tests and improves a socially mediated intervention that already has been shown to produce good engagement [24]. We believe that engagement happens because of the development of a sense of online community through extensive interpersonal and, especially, dyadic interactions [25,26]. In response to calls for the application of systems science to public health [27], we will apply network analysis to examine tie strength among buddies (paired people in the group based on similarity in age, gender, location, and education) [28] and to identify social brokers (people who facilitate communication between otherwise unconnected individuals) [29].

Our *first primary aim* is to test the 6-month efficacy of Tweet2Quit with biochemical verification of abstinence. We hypothesize that relative to control coed (male and female) groups (n=240), Tweet2Quit coed groups (n=480) will achieve significantly greater bioconfirmed sustained abstinence at 6-months follow-up. Our *second primary aim* is to test whether women do better in Tweet2Quit women-only versus coed groups. We hypothesize that women will achieve significantly greater bioconfirmed 6-month abstinence in Tweet2Quit woman-only groups (n=240) versus Tweet2Quit coed groups (n=240 women), as people tend to form stronger social connections with others who share their defining characteristics such as gender [30]. *Secondary aims* will test the same hypotheses but based on 3-month (end of treatment) sustained and bioconfirmed abstinence, and we will also test 7-day point prevalence abstinence at 1, 3, and 6 months.

Exploratory aims will study the Tweet2Quit groups’ social network structures with a focus on the buddy pairs and the identification of social brokers (group members who facilitate interaction between otherwise unconnected individuals), using both baseline theoretically based measures and observed tweeting behaviors. We predict that active buddies in the group will enhance tie strength, among both buddy-partner ties and partner-group ties, which will increase smoking abstinence. We also predict that better social brokers will enhance tie strength, as they will encourage more people to engage in the intervention (tweet their group), which will increase smoking abstinence.

## Methods

### Study Setting

The study setting is virtual—hosted online via the study website and Twitter—but the study is conducted by the University of California, Irvine (UCI).

### Trial Design

The study runs parallel groups, with treatment and control groups starting and stopping at the same time. Treatment group (n=20) and control group (n=10) allocation began once 30 participants passed screening, using a 2:1 ratio (see the Randomization section below).

### Inclusion Criteria

Participants are considered eligible if they are aged between 21 and 59 years (21 years being the strictest minimum age to receive nicotine replacement therapy [NRT] products in the United States); speak English, so they are able to communicate with their group; are current smokers (have smoked at least 100 cigarettes in their lifetime and are smoking at least five cigarettes a day) to meet the requirement for NRT products; have a mobile phone with an unlimited texting plan (unlimited data not required) and internet to receive intervention messages; have an active email account to receive quit-smoking guides; live in the continental United States, so that all participants are in a similar time zone to facilitate communication; can send/receive SMS text messages at least once a week; and have an active social media account to show they are familiar with and can communicate on this platform.

### Exclusion Criteria

Due to use of NRT in this study, participants are ineligible if they have contraindications for NRT including irregular heartbeat, high blood pressure not controlled with medication, recent heart attack, pregnant or breast feeding, skin allergies to adhesive tape, or serious skin problems [31]. Consistent with other quit-smoking trials, those who do not want to set a quit date or are not intending to quit in the next 30 days are excluded [31]. As this social media intervention uses a peer-to-peer support model, without a formal group moderator, and because this is an initial demonstration trial, individuals taking medicine for depression, using an illicit drug, or regularly using marijuana are excluded [32]. For study retention, personal contact information is required as is email verification. Those who fail to provide these contact details, or who provide nonworking phone numbers or emails, are excluded [33]. As we want to conduct saliva tests using webcams, that is, cameras on mobile phones, participants must show us they have this. To prevent problems with misrepresentation, those who fail the screening survey in the past are, henceforth, excluded. To prevent problems with contamination, we exclude those who already take medication for quitting smoking, who have participated in the 2011-2014 Tweet2Quit study [34], or who live with someone or have an immediate relative who has already participated or will participate in the current Tweet2Quit study.

### Electronic Cigarettes/Vaping

Electronic cigarettes (e-cigarettes)/vaping are discouraged during the course of the program but are not forbidden, and participants are not disqualified if these products are used. During the follow-up surveys, participants are asked about the use of e-cigarettes/vaping to include in the analyses.

### Treatment (Twitter) Arms: Coed and Women-Only

The treatment participants are put into a private Twitter group of 20 participants as well as paired with another participant in the group for a buddy system. These participants are encouraged to tweet (message) each other daily for support by logging into a Twitter account that we provide to them free of charge, and they receive a daily discussion topic as a tweet to help start conversations. The discussion topics encourage participants to get to know each other, express their quitting goals, and share tips on how to stay smoke free. Participants are encouraged to send at least one tweet a day, and their participation is monitored automatically by the program.

In addition, treatment participants receive daily automated texts providing feedback on their prior-day tweeting behavior, which praises tweeters and encourages nontweeters to engage. If the number of tweets over consecutive days falls below the minimum expected (varies per week; decreases as the group goes on), an additional text is sent to get participants onto Twitter to respond to a new discussion topic. These extra tweets and texts are to encourage treatment participants to interact with other group members about quitting.

Coed treatment groups consist of 20 participants with a mixture of men and women. However, there are even numbers of men and women in each coed treatment group, so that buddies can be of the same gender. Women-only treatment groups have 20 participants consisting of only women. All treatment interventions (eg, discussion topics) for the coed and women-only groups are the same. All treatment participants receive 8 weeks of nicotine patches (1 per day) and their choice of gum or lozenges (12 per day while on the patch), and Smokefree.gov quit guides are emailed about every 5 days.

### Control Arm

Control participants receive 8 weeks of nicotine patches (1 per day); their choice of nicotine gum or lozenges (12 per day while on the patch); and Smokefree.gov quit guides, which are emailed about every 5 days. Treatment and control groups run parallel to each other, ie, start at the same time.

### Withdrawal

Participants can drop from the study, by request, at any time. If a participant stops sending tweets to their group, the team still follows up at the scheduled times unless otherwise requested, and the participants are still eligible for the gift cards for completing follow-up assessments.

### Mode of Delivery/Communication

The NRT products are provided via mail, but all other components of the intervention are provided over the internet or email. Each participant receives log-in information, as well as all resource guides, automatically via email. Daily reminders are sent via SMS text messages. If the participant needs to interact with the study team, they can send an email (a contact form is available on the study website) or call the staff directly. The support group is virtually located on Twitter, and participants only have to log into their accounts to interact with their support group. Guides that clearly show participants how to log in and use Twitter are provided and also posted on the study website.

### Outcomes

#### Primary Outcome

Participants report their use of tobacco products at 1, 3, and 6 months after the quit date (the date they selected to quit smoking) by answering the following questions: “How many cigarettes have you smoked,” “How many other tobacco products have you used,” and “How many times have you used e-cigarettes since the quit date.” If participants self-report no tobacco use and no use of NRT, this is confirmed biochemically using a salivary cotinine test at 3 and 6 months after the quit date. Participants will complete the salivary test while we observe on a webcam (camera on their mobile phone), they take a picture of the test results, and they text us the picture. If webcam observation is unfeasible, participants will complete the salivary test on their own and text us a picture of the results. We will apply the Russell Standard for sustained abstinence, allowing five or fewer instances of tobacco use over 6 months. We will use online self-reported surveys to collect the tobacco use data.

In our primary (most rigorous) analysis, we will consider a cotinine-positive test, regardless of source, to be nonabstinent. In additional analyses, we will code as abstinent those who assert tobacco abstinence but continue use of US Food and Drug Administration–approved NRT. In other analyses, we will consider use of electronic nicotine delivery systems (ENDS; such as e-cigarettes or vaping), with ENDS users coded first as nonabstinent and then for comparison as abstinent. Secondary analyses will examine 3-month (end of treatment) sustained and bioconfirmed abstinence and 7-day point prevalence abstinence at 1, 3, and 6 months.

### Time Schedule of Enrollment and Participation

Table 1 shows the study flow for each cohort. Enrollment and allocation to condition occurred first, followed by the 3-month intervention, with assessments occurring at 1 month, 3 months, and 6 months.

**Table 1:** Study flow.

Study activity		Study period and timepoint							
		Enrollment	Allocation	Post allocation					Close-out
		Month 0	Month 0	Month 1	Month 2	Month 3	Month 4	Month 5	Month 6
**Enrollment**									
	Eligibility screen	✓							
	Informed consent	✓							
	Verification of email, phone, and webcam	✓							
	Randomization		✓						
**Treatments**									
	Twitter mobile support group			✓	✓	✓			
	Nicotine replacement therapy			✓	✓	✓			
	Emails linked to Smokefree.gov			✓	✓	✓			
**Assessments**									
	Follow-up surveys			✓		✓			✓
	Saliva tests					✓			✓

### Recruitment

All recruitment is done online through advertisements. Primarily, Facebook ads are purchased to recruit participants. Ads are designed to target people on Facebook who indicate interest in smoking or quitting smoking, with additional ZIP code targeting of black/African American participants and Hispanic/Latino participants living in high-smoking areas and some limited targeting of Spanish-English bilingual speakers to increase participant diversity. People see a study ad on Facebook, and if they click on the ad, they are brought to the study website, where they can fill out a brief form indicating interest. In addition, some free Google Ads (provided through the Google Grants program to assist nonprofits) appear for the study when quit-smoking keywords are entered into the Google search engine. Finally, an ad listed on Smokefree.gov directs traffic to the study website.

### Randomization

Randomization occurs once the targeted number of participants for a cohort is reached based on the online eligibility screen and the verification of email, phone, and webcam (camera on mobile phone) for saliva testing. Participants are randomized to the treatment or control condition by statistician KD using deidentifiable ID numbers. The allocation is fully random, with each person having an equal chance of ending up in the treatment or control condition. Whether the cohort treatment is coed or women only is also randomly determined by KD. After the treatment commences, the condition assignment is not blinded to participants and the research team; however, during data collection, the staff members that contact participants (if participants do not complete follow-up surveys on their own) are not told the study condition of participants to ensure equal effort in gathering data. The process of starting a new treatment/control cohort is repeated every few weeks to a month until 960 participants are attained (36 treatment/control cohorts beyond the 20-person pilot).

### Consent

As the study is conducted via internet across the continental United States, rather than in person, a waiver of signed consent has been obtained. At the beginning of screening, potential participants receive an information sheet that contains study information, and they must consent (by clicking yes or no) to continue with screening.

### Institutional Review Board

The study is under the oversight of the UCI’s and the Stanford University’s institutional review boards (IRBs). Any requested changes to the protocol are submitted through these IRBs.

### Study History

During 2011 to 2014, we ran an initial evaluation of Tweet2Quit that tested this mobile support group platform for quitting smoking, and we observed a self-reported abstinence rate of 40% at 60 days compared with 20% in the control group [34]. This study extends follow-up to 6 months to document sustained abstinence, uses bio-confirmation to validate self-report, and incorporates a women-only Tweet2Quit treatment arm. We also introduce demographically matched buddy pairs for extra support within the group, tweet streaks to encourage daily dyadic interactions (daily text messages to participants, showing the number of days in a row they tweeted other participants without missing), and low tweet messaging that supplies extra topics when the number of group tweets is low. We added these features to the Tweet2Quit intervention because we observed that tweeting about relevant topics, such as using nicotine patches and countering roadblocks to quitting, increased the participants’ chance of achieving abstinence [24].

### Revisions

This study is based on the most recent protocol version, September 2018. During the course of the study, there were no major study revisions, but we increased the monetary incentives for completing the outcome assessments to improve response rates.

### Data Collection, Management, and Analysis

Self-assessment follow-up surveys are automatically emailed to participants by the software program Qualtrics (Provo, Utah) at 1, 3, and 6 months after a participant’s quit date (the date they set to quit smoking). These surveys are compliant with the Checklist for Reporting Results of Internet E-Surveys. Participants’ responses to the online survey questions are kept on the Qualtrics server until they are downloaded by the study team for analysis. When participants do not complete a survey themselves, the research team reaches out to them via phone to complete the survey with them, and the research team enters the data for them into the online Qualtrics survey. The survey questions assess the effectiveness of the intervention at promoting abstinence based on participants’ self-reports, and they also track participants’ self-reported use of NRT and ENDS products. All participants receive these surveys, along with a monetary gift card for completion, regardless of condition and even if the participant does not actively participate in (tweet) their treatment group.

### Statistical Analyses

Dropout rates are examined by condition. Every effort is made to limit the amount of missing data from survey attrition by doing persistent follow-up with participants and contacting their preidentified collaterals (family members and friends) to urge survey completion. Before analysis, we will examine baseline predictors of attrition. If it appears that attrition is related to measured aspects of the participants, we will include those measures as covariates in the models. Sensitivity analyses will check that the methods of dealing with missing data do not have a major impact on the results. We will repeat the attrition analyses under two different models, one in which the missing data are assumed to be positive for smoking and one using only the respondents who did not have missing data. We expect to find that although estimates may change some, the conclusions generated by the modeling should not change.

For primary hypothesis testing of sustained abstinence at 6 months, we will use the 6-month postquit date survey (based on the quit date selected by the participant at the beginning of the study) and biochemical salivary cotinine verification. To test the *primary aim 1 hypothesis*, analyses will compare sustained abstinence for participants randomized to coed Tweet2Quit treatment versus control. Sustained abstinence will be modeled as a function of study arm (coed Tweet2Quit or control), gender, cohort, and individual using a logistic model and generalized estimating equations (Proc Genmod in SAS; SAS Institute Inc, Cary, North Carolina) to account for the clustering of individuals within cohort. Our statistical methods will use all the data in parameter estimation. We will test the coefficient of the study arm or treatment condition parameter by gender, if there is a treatment condition by gender interaction.

The same type of generalized logistic model, but focusing on women, will be used to test the *primary aim 2 hypothesis*, on whether women will achieve greater 6-month sustained, bioconfirmed abstinence if randomized to a women-only versus coed Tweet2Quit treatment group. Secondary hypotheses concerning sustained abstinence at 3 months (ie, at treatment end) and point prevalence (prior 7 days) abstinence at 1, 3, and 6 months will be tested using the same modeling approaches.

Exploratory aims will be met by testing our mediational models of predictors of abstinence. Regression models of each individual path will be tested. Subsequently, using a structural equation modeling approach, we will test whether the relationships between buddy pairs and abstinence, and social brokers and abstinence, can be accounted for by tie strength or other network characteristics, with direct paths modeled as well.

### Smokefree.gov Collaboration

Whenever treatment or control participants receive our email with a link to a Smokefree.gov quit guide and click on the link, their behavior on the Smokefree.gov website, including the number of page views and time spent, is automatically logged. Smokefree.gov provides us with these data on usage of their quit-smoking guides so that we can determine if guide usage improves abstinence and if the Tweet2Quit treatment affects guide usage.

### Data and Safety Monitoring Board

As the interventions are fairly standard and low-risk, a data safety monitoring board is not used.

### Confidentiality

All collected data are kept on secure online databases that are password protected with access limited to the study team. Subject-identifiable data will be retained until publication, at which point it will be destroyed. At the end of each group, Twitter profile pictures (pictures of participants’ faces that we use to create their study accounts on Twitter) will be destroyed. Pictures of saliva test results, which do not show subject-identifiable data, will be kept indefinitely as proof of abstinence.

### Privacy

Privacy is a concern with any study that is conducted online, especially in a support group that encourages participants to be open with each other. Although social media is a public platform, there are Twitter settings that quickly ensure groups are permanently and completely private. All these privacy features are turned on to prevent participants from being easily searched on Twitter or have their tweets seen by their family, friends, or other people outside the treatment group. Their tweets are even hidden from other treatment groups in the study. Participants are further taught how to interact on the Twitter platform to keep themselves and others safe and the group private.

### Access to Data

UCI will be working with personnel from both the University of California, San Francisco (UCSF), and Stanford University in the analysis portion of this study, sharing both the survey data and the bioconfirmed abstinence data. UCI personnel will remove all identifiable data from the files before sharing with the other sites. JP has secured IRB approval from Stanford University and will be working in collaboration with UCI. No subject enrollment or data collection has been conducted at Stanford. Data will be shared between Stanford and UCI primarily in a deidentifiable fashion, but some datasets may include minimal identifiable data (eg, Twitter usernames). JP receives subject-identifiable data from UCI in the event of a serious adverse event but does not receive identifiable data at any other time. In sum, both deidentifiable data and identifiable data will be seen by JP while working from Stanford.

KD will conduct his portion of the study from UCSF; however, he will not have access to identifiable data. KD assists in randomizing participants to groups, but he only uses deidentifiable subject IDs for randomization and data analysis. KD has no interaction with human subjects. Both JP and KD will assist in analysis of deidentifiable data.

### Dissemination of Data

The study team plans to present the findings at academic conferences and publish them in peer-reviewed academic journals. In addition, the team plans to discuss the findings with government officials such as those affiliated with Smokefree.gov. Only research team members will be authors of any papers written, and participant identifiers will be removed.

## Results

### Results for Recruitment and Retention

IRB approval of the study began in March 2016, with National Institutes of Health funding running from March 2016 to March 2021. Recruitment has taken place from November 2016 to September 2018, and 980 participants have been enrolled. However, 20 of these participants were part of a pilot group to pretest the intervention and will not be used in the main analyses. As part of the randomized controlled trial (n=960), 480 participants have been allocated to the coed treatment condition, 240 participants have been allocated to the women-only treatment condition, and 240 participants have been allocated to the control condition. Table 2 provides the demographics of these participants.

**Table 2:** Demographics of participants in the randomized controlled trial by condition.

Intervention		Coed treatment (n=480)	Women-only treatment (n=240)	Control (n=240)
Mean age, years (SD)		39.0 (9.52)	39.8 (9.45)	40.4 (9.69)
**Gender, n (%)**				
	Male	142 (29.58)	0 (0)	73 (30.42)
	Female	338 (70.42)	240 (100)	167 (69.58)
**Ethnicity, n (%)**				
	White	376 (78.33)	204 (85.0)	197 (82.08)
	African American/black	55 (11.46)	22 (9.17)	21 (8.75)
	Hispanic/Latino	21 (4.38)	6 (2.50)	10 (4.17)
	Other	28 (5.83)	8 (3.33)	12 (5.0)

Only 8 (<1%) participants withdrew from the study, that is, they requested removal from all intervention emails and texts (eg, the Smokefree.gov quit guides) and all assessments, and if in a treatment condition, they dropped out of their Twitter support group. At 6-month follow-up (the primary outcome measure), we have collected 896 surveys or 93.3% (896/960), with 56 of the participants or 5.8% (56/960) lost to follow-up and 8 or <1% (8/960) who discontinued the intervention (see Multimedia Appendix 1 for the breakdown at each follow-up). The primary analysis will compare the focal conditions to assess the percentages of participants with biometrically verified sustained abstinence. For this primary analysis, we have collected data from 437 of the 480 participants (91.0%) in the coed treatment condition, 226 of the 240 participants (94.2%) in the women-only treatment condition, and 233 of the 240 participants (97.1%) in the control condition.

No serious adverse events have occurred. There have been no security breaches, but there was a technical error that caused the feedback texts and tweet streaks to be inaccurate for a few days in one group. Participants were told about the technical error and continued to interact normally.

### Power Analysis

Sample sizes were selected to have sufficient power to test both primary hypotheses. For testing the aim 1 hypothesis, a logistic regression to assess power, based on sustained abstinence estimates of 20.0% for Tweet2Quit-coed (n=480) versus 8.0% for usual care-coed (n=240), estimated 98% power at *P*=.05 and 97% power with 13% (94/720) expected survey attrition. For testing the aim 2 hypothesis, a logistic regression to assess power, based on sustained abstinence estimates of 26.0% for Tweet2Quit-women-only (n=240) versus 14.0% for women in Tweet2Quit-coed (n=240), estimated 88% power at *P*=.05 and 83% power with 13% (62/480) expected survey attrition.

Regarding the structural equations models we will use to test for process effects, the standard fit statistics will be evaluated. We will not rely on the Chi-square statistic as it is sensitive to sample size. If we find poor fit in this exploratory analysis, we will examine the individual parts of the model to determine what aspects indicate poor measurement or relationships and revise the model accordingly.

## Discussion

### Principal Findings

This study builds on the encouraging effects found with the Tweet2Quit quit-smoking platform, which was developed and tested from 2011 to 2014. Current extensions include an analysis of 6-month sustained bioconfirmed abstinence and a comparison of women-only and coed treatment groups. Existing quit-smoking programs utilize Twitter groups or daily texts, but our program combines these ideas and creates private Web-based groups that connect people across the United States to receive peer support and daily messages for quitting smoking. We will test if after 6 months the participants attain sustained abstinence. Participants in the coed control condition and the coed Tweet2Quit condition will be compared. In addition, women participants in the coed Tweet2Quit condition and the women-only Tweet2Quit condition will be compared.

### Strengths and Limitations

As only those who regularly use social media can participate in our Tweet2Quit program, a concern could be raised about reaching the relevant US population. However, this concern is mitigated by the fact that 73% of US adults who are online use social media, so we feel that we can reach the majority of the online population [12].

In our study, there is also the concern that, because participants know which condition they are in, if placed into the control rather than a treatment condition, they may withdraw from the study or its assessments. However, less than 1% of participants withdrew from the study. In addition, we have a team follow-up with all the participants who do not complete a survey on their own to gather the data. As a result, we have attained similar response rates across the study conditions and 90% or higher response rates overall.

### Conclusions

If the Tweet2Quit treatment is found to be efficacious, it will provide an easily accessible program for anyone nationwide to help them quit smoking and stay smoke free. Furthermore, the intervention can be easily replicated in other health contexts because the Twitter platform can be used free of charge in nearly the entire world.

## Appendix

Multimedia Appendix 1 CONSORT diagram for the randomized controlled trial.

Multimedia Appendix 2 Peer-reviewer report from the NIH.

## Abbreviations

e-cigarette: electronic cigarette
ENDS: electronic nicotine delivery systems
IRB: institutional review board
NRT: nicotine replacement therapy
UCI: University of California, Irvine
UCSF: University of California, San Francisco
