# Supplementary material for: The Use of Web-Based Support Groups Versus Usual Quit-Smoking Care for Men and Women Aged 21-59 Years: Protocol for a Randomized Controlled Trial
Source: JMIR Res Protoc. 2020 Jan 14;9(1):e16417. doi: 10.2196/16417 (PMC6996731; doi:10.2196/16417)
Supplement: Multimedia Appendix 1 [file resprot_v9i1e16417_app1.docx]

**Multimedia Appendix 1. Consort Diagram**

**Enrollment**

Assessed for Eligibility (n=4,589)

**Excluded n=3,629 (79.1%)**

Did not meet 1+ inclusion criteria

- Didn’t Finish Screening Survey n=1,257 (29.6%)
- Didn’t Consent n=790 (18.6%)
- Health Contraindicated n=682 (16.1%)
- Failed/Didn’t do Webcam/Phone/Email Verification n=639 (15.0%)
- Daily Marijuana or Any Hard Drug Use n=302 (7.1%)
- On Smoking Cessation Drugs or NRT n=127 (3.0%)
- Less than 5 cigs/day n=100 (2.4%)
- No Access to Internet n=99 (2.3%)
- Other n=252 (5.9%)

**Randomized in 36 cohorts n=960 (20.9%)**

No participants were excluded from the analyses, but response rates were lower with sustained (vs. 7-day) abstinence when participants refused follow-up questions.

**Follow-Up at 3 Month Post-Quit Date**

**Follow-Up at 6 Month Post-Quit Date**

**Allocation**

**Follow-Up at 1 Month Post-Quit Date**

**Analyzed**

n= 228 (95.0%)

**Lost to Follow-up**

n= 12 (5.0%)

**Discontinued Intervention**

n=0

**Analyzed**

n= 222 (92.5%)

**Lost to Follow-up**

n=17 (7.1%)

**Discontinued Intervention**

n=1 (0.4)

**Analyzed**

n= 429 (89.4%)

**Lost to Follow-up**

n= 50 (10.4%)

**Discontinued Intervention**

n=1 (0.2%)

**Analyzed**

n= 434 (90.4%)

**Lost to Follow-up**

n= 46 (8.3%)

**Discontinued Intervention**

n=6 (1.3%)

**Analyzed**

n= 219 (91.3%)

**Lost to Follow-up**

n= 19 (7.9%)

**Discontinued Intervention**

n=2 (0.8%)

**Analyzed**

n= 223 (92.9%)

**Lost to Follow-up**

n= 17 (7.1%)

**Discontinued Intervention**

n=0

**Analyzed**

n= 233 (97.1%)

**Lost to Follow-up**

n= 7 (2.9%)

**Discontinued Intervention**

n=0

**Analyzed**

n= 437 (91.0%)

**Lost to Follow-up**

n= 37 (7.7%)

**Discontinued Intervention**

n=6 (1.3%)

**Analyzed**

n= 226 (94.2%)

**Lost to Follow-up**

n= 12 (5.0%)

**Discontinued Intervention**

n=2 (0.8%)

**Allocated to Coed Intervention n=480 (50.0%)**

- Nicotine patches and gum or lozenges
- Smokefree.gov website links
- Tweet2Quit co-ed groups of 20

**Allocated to Women-Only Intervention n=240 (25.0%)**

- Nicotine patches and gum or lozenges
- Smokefree.gov website links
- Tweet2Quit women-only groups of 20

**Allocated to control n=240 (25.0%)**

- Nicotine patches and gum or lozenges
- Smokefree.gov website links
