# Supplementary material for: The Use of Web-Based Support Groups Versus Usual Quit-Smoking Care for Men and Women Aged 21-59 Years: Protocol for a Randomized Controlled Trial
Source: JMIR Res Protoc. 2020 Jan 14;9(1):e16417. doi: 10.2196/16417 (PMC6996731; doi:10.2196/16417)
Supplement: Multimedia Appendix 2 [file resprot_v9i1e16417_app2.pdf]

SUMMARY STATEMENT
(Privileged Communication)

PROGRAM CONTACT:
Wen-Ying Chou
(240)276-6954
chouws@mail.nih.gov

Release Date: **11/04/2015**

Application Number: **1 R01 CA204356-01**

Principal Investigators (Listed Alphabetically):
PECHMANN, CORNELIA PHD (Contact)
PROCHASKA, JUDITH J. PHD

Applicant Organization: UNIVERSITY OF CALIFORNIA-IRVINE

Review Group: **IPTA**
Interventions to Prevent and Treat Addictions Study Section

Meeting Date: **10/08/2015**
Council: **JAN 2016**
Requested Start: **04/01/2016**

RFA/PA: **PA13-302**
PCC: **W3CI**
Dual PCC: **CM/TAW**
Dual IC(s): **DA**

Project Title: **Social Media Technology for Treating Tobacco Addiction**

SRG Action: **Impact Score: 15 Percentile: 2**
Next Steps: **Visit http://grants.nih.gov/grants/next_steps.htm**
Human Subjects: 30-Human subjects involved - Certified, no SRG concerns
Animal Subjects: 10-No live vertebrate animals involved for competing appl.
Gender: **1A-Both genders, scientifically acceptable**
Minority: **1A-Minorities and non-minorities, scientifically acceptable**
Children: **1A-Both Children and Adults, scientifically acceptable**
Clinical Research - not NIH-defined Phase III Trial

Project Year	Direct Costs Requested	Estimated Total Cost
1	499,991	755,092
2	499,882	754,927
3	499,949	755,029
4	499,380	754,169
5	497,693	751,622
TOTAL	2,496,895	3,770,839

ADMINISTRATIVE BUDGET NOTE: The budget shown is the requested budget and has not been adjusted to reflect any recommendations made by reviewers. If an award is planned, the costs will be calculated by Institute grants management staff based on the recommendations outlined below in the COMMITTEE BUDGET RECOMMENDATIONS section.

1R01CA204356-01 Pechmann, Cornelia

ADMINISTRATIVE NOTE

RESUME AND SUMMARY OF DISCUSSION: This application proposes to revise, pilot test, and evaluate in a randomized trial the “Tweet2Quit” social media intervention for smoking cessation. This intervention is one of the first real-time, fully automated, twitter-based interventions. Both the environment and investigators are excellent. Other strengths included: excellent pilot data from a previous R34 in support of the application and was useful for designing the current trial, careful basis in current literature, focus on gender differences in response to smoking cessation interventions, and sophisticated analysis of online social dynamics. In summary, the committee concluded that the potential overall impact was high.

DESCRIPTION (provided by applicant): Social Media Technology for Treating Tobacco Addiction. The internet was built for communication. This research project, with its cutting-edge social network approaches and analyses, will examine the potential of the internet, and more specifically social media, to engage health communications for quitting smoking and preventing relapse for addiction treatment and cancer prevention. Further, the design tests the personalized benefits and treatment processes of a women-centered internet cessation intervention. With initial funding from an NIH R34 innovation award, we developed and evaluated a novel online social media intervention for tobacco cessation called Tweet2Quit. Tweet2Quit is a 100-day online intervention that uses a novel, low cost, automated approach to encourage and direct peer-to-peer social support for quitting smoking. Private Twitter groups of 24 smokers are set up, and engagement is encouraged and directed by daily auto feedback and daily auto messages that suggest discussion topics consistent with US treatment guidelines for tobacco use cessation. In a randomized controlled trial (N=160), all participants received advice to quit, referral to the NCI Smokefree.gov website, and 8 weeks of nicotine patch (“usual care”); and half were then randomized to a Tweet2Quit peer support group to test the net benefit of the social media intervention. At 60-days follow- up, Tweet2Quit participants reported 40% sustained abstinence compared to 20% for controls ($p=.017$), and engagement in the intervention was related to abstinence ($p<.001$). Consistent with the tobacco treatment literature, in both study conditions, men were more likely to quit smoking than women, and the gender effect in Tweet2Quit was moderate ($d=.55$). Notably, women in Tweet2Quit tweeted as much as the men, though the content differed thematically. The proposed randomized controlled evaluation (N=960) of the Tweet2Quit intervention will biochemically verify abstinence out to 6-months follow-up and will test the personalized benefit for women of a women-only versus co-ed Tweet2Quit group. In a 3-arm design, we will compare: 1) usual care, 2) Tweet2Quit-coed, and 3) Tweet2Quit-women only. Each 24-person Tweet2Quit group will include 12 buddy pairs based on similarity in demographics and abstinence goals. Also automated pattern detection will identify dips in tweeting and trigger automated alerts and engagement strategies. Our primary aims test hypothesis 1: Relative to usual care (n=240), Tweet2Quit-coed groups (n=480) will achieve significantly greater bioconfirmed sustained abstinence out to 6-months follow-up, and hypothesis 2: Women in Tweet2Quit will achieve significantly greater bioconfirmed 6-months abstinence in woman-only groups (n=240) vs. coed groups (n=240 women). Our secondary aims are to test the same hypotheses based on 3-month (end of treatment) sustained abstinence and 7-day point prevalence at 1, 3, and 6 months. Exploratory aims will study the Tweet2Quit groups' social network structures with a focus on the identification of buddy pairs and social brokers and test if these relationships are stronger for women in women-only groups versus women in coed groups of Tweet2Quit.

PUBLIC HEALTH RELEVANCE: Social Media Technology for Treating Tobacco Addiction Tweet2Quit is a low cost, web-based, social media-enabled, peer-to-peer intervention for smoking cessation with fully automated setup and automated daily texts to encourage peer discussions. In a randomized

controlled trial, we aim to determine intervention efficacy out to 6 months follow-up with bioconfirmation of abstinence and to assess the benefit among women of a women-only versus coed social media peer-to-peer group. Findings will inform observed gender differences in cessation and the use of social media for low cost, scalable addiction interventions. 1

CRITIQUE 1

Significance: 1
Investigator(s): 1
Innovation: 1
Approach: 3
Environment: 1

Overall Impact: Following very promising data collected as part of an R34, the proposed RO1 randomized controlled trial will test the Tweet2Quit intervention efficacy, including biochemically verify abstinence out to 6-months, in a 3-arm design: usual care, Tweet2Quit-coed, and Tweet2Quit-women only. Impact is very high given low cost and sustainability of this intervention, with auto messaging as opposed to peer moderators. Innovation is high via delivery on Twitter, including matching of peer dyads via computer algorithm and examination of social brokers (who will be assigned to each group). The examination of a women only condition, as opposed to a mixed gender condition, increases significance given the reduced quit rates among women. The investigative team is well suited to achieve study aims. The approach is strong, using a comparative efficacy trial in which the active control condition will receive online smoking cessation website with patch/gum and biological confirmation of self-report. The weaknesses are minor, with the exception that outcomes are relatively short term (6 months). Nonetheless, this application is very strong, with high impact.

1. Significance:

Strengths

- Testing use of social media, specifically Twitter, to facilitate social support for smoking cessation has high significance by ease of delivery, low cost and high reach.
- Convincing argument that could be scalable and replicated for other health behaviors (e.g., alcohol, other drugs), which increases significance of the approach.
- Although unlikely that Twitter would disappear in the near future, the intervention could be delivered on other social media platform such as Facebook.
- Instead of relying on peer leaders, the 100 day program uses auto messages to increase interaction which has high sustainability if efficacious.

Weaknesses

- Daily use of Facebook (eligibility criteria) limits future impact; however 70% use Facebook daily.

2. Investigator(s):

Strengths

- MPI: Dr. Pechmann is a professor of Marketing at Vanderbilt with training in psychology and has conducted research on tobacco control issues for two decades. Although she has never been the PI on a R01, she was the PI of the R34 that developed and piloted the Tweet2Quit intervention which increases confidence in her ability to lead this study.

- MPI: Dr. Prochaska at UCSF has experience with intervention studies and will lead the team in social media recruitment; also, as a clinical psychologist, she will monitor group interactions.
- MPI plan has clear roles, with Dr. Pechmann leading the technology pieces and Dr. Prochaska leading the intervention, recruitment and retention pieces.
- Roles are mostly distinct with appropriate expertise: Dr. Lakon (Co-I) will lead social network analysis, Dr. Ramo, (consultant) will assist with the social medial recruitment; Dr. Delucchi (consultant) will prepare the randomization scheme and analytic strategy; and Dr. Sanders-Jackson (co-I) will conduct social network analytics.

Weaknesses

- None noted

3. Innovation:

Strengths

- Using Twitter (social media) to deliver low-cost and high reach interventions by private social support groups with technology matched peers (using a prior tested computer algorithm that increases dyad ties) is highly innovative.
- Inclusion of mixed gender group and a women only group is highly innovative and important given lower quit rates among women.
- Prescreening will result in identification of "social brokers" (personality trait of self-monitoring; greater centrality in social network analyses) who in the pilot posted more tweets and had great abstinence; these participants will be balanced by groups (24 per group) to increase likelihood of interaction and success across groups. This approach is highly innovative.
- Exploratory social network analyses will compare peers, social brokers, and examine interactions in the Twitter group is interesting and novel.
- Use of pilot data regarding types of Tweets in relation to engagement and outcome will improve the proposed revised intervention.

Weaknesses

- None noted

4. Approach:

Strengths

- Self-report of quitting augmented with biochemical data collection via the mail with Skype verification.
- Careful plan for participant identify verification via IP addresses as well as video/skype with participants at enrollment and during saliva tests.
- Promising pilot data currently in review (e.g., doubled sustained reported abstinence relative to usual care at 2-month follow-up, with high engagement which was related to abstinence); however, the quit rates were lower in women than men.
- Enhancement of prior intervention by linkage with a peer buddy and auto messages to increase engagement; although this approach has face validity and appears to be a strength, these enhancements are untested.
- Rigorous control condition (smokefree.gov + patch/gum).

- 10 cohorts of 96 assigned to 3 conditions will allow for adequate power.
- Detailed theory driven hypotheses for models testing mediation via social network analyses.

Weaknesses

- Short term follow-up (1, 3, 6 months) is moderate limitation in that data will be lacking on sustainability of quitting.
- 90% 2 month follow-up (with abbreviated assessments for hard to reach) is good, with planned monetary incentives at 6 months; however, demonstration of ability to retain participants with the full assessment at 6 months is unclear.
- Prior study resulted in few minority participants; thus, the team now includes a consultant with expertise in Facebook recruitment including increasing minority participation.
- Because there will not be a therapist moderator, daily marijuana use is described as an exclusion criterion (only 21 smokers excluded); this rationale is not convincing.
- The lack of measurement of alcohol, marijuana, and other substances seems like a missed opportunity.

5. Environment:

Strengths

- Facilities at University of California Irvine are sufficient to conduct the proposed research in terms of space and computing etc., with access to research staff through the undergraduate research opportunity program.
- Letter of support with detailed intervention costs from the computer development firm Web Advanced, increases confidence in ability to develop the proposed intervention.
- Letter of support from NCI Smokefree.gov, with ability to share data on visits to the site, which is a strength.

Weaknesses

- None noted

Protections for Human Subjects:

Acceptable Risks and/or Adequate Protections

New names/Twitter accounts will be created for confidentiality.

Data and Safety Monitoring Plan (Applicable for Clinical Trials Only):

Acceptable

Overall acceptable; however, the timeframe for reporting SAEs is needed.

Inclusion of Women, Minorities and Children:

- Sex/Gender: Distribution justified scientifically
- Race/Ethnicity: Distribution justified scientifically
- Inclusion/Exclusion of Children under 21: Including ages < 21 justified scientifically
- Ages 18-59 with justification for inclusion of youth ages 18-20 and exclusion of illegal smokers under the age of 18.

- Women oversampled to allow for women only arm of study.
- Minorities will be oversampled (than represented on Facebook) to 35%, which is strength.

Vertebrate Animals:

Not Applicable (No Vertebrate Animals)

Biohazards:

Not Applicable (No Biohazards)

Resource Sharing Plans:

Acceptable

Budget and Period of Support:

Recommend as Requested

CRITIQUE 2

Significance: 2
Investigator(s): 1
Innovation: 2
Approach: 3
Environment: 1

Overall Impact: This is very well-written grant application proposing to further test a peer-to-peer, twitter group intervention based on highly informative data from a previous R34 grant. The investigators are highly experienced and knowledgeable as evidenced by the detailed and thoughtful application. The pilot data are rich and have led to a separate aim to improve the gender inequity in post-treatment smoking cessation rates, as well as innovative mechanisms related to matching dyad pairs and social leaders emerging within each twitter group. The main weakness that somewhat lessens the impact potential is the higher educated/SES sample that will result from the inclusion/exclusion criteria and the nature of the intervention itself. It is also unfortunate that the biochemical validation was not tested during the R34 study. Weaknesses do not outweigh strengths of this excellent application, however. The intervention, if efficacious, has the potential for a relatively large impact among smoking populations who regularly use mobile phones and social media.

1. Significance:

Strengths

- Social media is increasingly popular and is an important platform on which to reach and engage smokers.
- The intervention is fully automated and low cost which increases potential for implementation/dissemination. An NCI Program Director has offered support for disseminating the program on the Smokefree.gov website if found to be efficacious.
- Determining ways in which to reduce gender inequity in quit rates is highly significant.

Weaknesses

- Targets educated, middle/upper class populations with smartphone and/or computer for on-line access, e.g., 30 – 40% of pilot participants had a college degree. Most smoking is concentrated in lower SES populations that may not have access to such resources.

2. Investigator(s):

Strengths

- An outstanding team of investigators with diverse areas of expertise, such as marketing, health communication, smoking cessation, mobile and web-based interventions, network analyses, psychology, has been assembled.
- Multiple PIs are proposed and justified; each with distinct yet complementary backgrounds and experience.

Weaknesses

- None noted.

3. Innovation:

Strengths

- The proposed Tweet2quit intervention is one of the first real-time, fully automated, twitter-based interventions
- The intervention explores novel mechanisms to promote change, including the utility of matched buddy pairs (dyadic tie strength) and “social brokers” (betweenness centrality) in increasing sustained cessation.

Weaknesses

- Social network-based interventions are not completely novel but research in this area is in its infancy making this an important study.

4. Approach:

Strengths

- Excellent pilot data from a previous R34 is presented in support of the application and was highly informative for designing the current trial.
- The design allows a test of the overall intervention in a male and female sample vs. usual care, as well as a third group for testing a manipulation targeting women (women only groups vs. co-ed twitter groups).
- Both discussion topic and engagement feedback messages will be sent to participants with early detection of nonparticipation via website-based pattern detection processes, which will trigger additional prompts to encourage re-engagment.
- Data assessing engagement from the smokefree.gov website will also be collected.
- Outcomes follow recommended guidelines for smoking cessation trials and include multiple measures.
- Biochemical validation is proposed which is challenging (but necessary) for large media-based interventions.
- False reporting rates are taken into account in the power analysis.

Weaknesses

- Study methods require participants to have a smartphone and/or a computer, reducing diversity in SES/level of functioning and potential for generalization to lower income populations who tend to smoke at higher rates.
- In the pilot data analysis, nonrespondents were counted as missing but the % of nonrespondents was not reported.
- Biochemical data collection via web or phone camera and mailed cotinine test strips has not been fully tested, although there is a plan to gain experience in an initial pilot phase.
- Requiring daily Facebook use seems a little heavy-handed and is based on correlational data that daily Facebook users had more tweets. It could be that these participants were more interested in social media via multiple platforms and not that the use of Facebook caused more tweeting.
- Effect size estimates for sustained abstinence at 6 months in the women only twitter group are proposed to be equal to the sustained abstinence rates of men at 60 days in the co-ed developmental study. This seems overly optimistic.

5. Environment:

Strengths

- Outstanding resources are available to support the proposed study at the institutions of both PIs.

Weaknesses

- None noted.

Protections for Human Subjects:

Acceptable Risks and/or Adequate Protections

- Risks are minimal and addressed appropriately. Appropriate precautions are in place.

Data and Safety Monitoring Plan (Applicable for Clinical Trials Only):

Acceptable

- A satisfactory plan has been developed.

Inclusion of Women, Minorities and Children:

- Sex/Gender: Distribution justified scientifically
- Race/Ethnicity: Distribution justified scientifically
- Inclusion/Exclusion of Children under 21: Including ages < 21 justified scientifically
- Men and women, all races/ethnicities, and children between the ages of 18 - 21 are included.

Vertebrate Animals:

Not Applicable (No Vertebrate Animals)

Biohazards:

Not Applicable (No Biohazards)

Budget and Period of Support:

Recommend as Requested

CRITIQUE 3

Significance: 1
Investigator(s): 3
Innovation: 1
Approach: 2
Environment: 1

Overall Impact: This application seeks to revise, pilot test, and evaluate—in a fully powered randomized trial—the fully automated "Tweet2Quit" social media (Twitter-based) intervention for smoking cessation. If successful, this trial could validate the efficacy of a highly replicable, accessible, high-reach intervention for smoking cessation that offers a unique new option for those wishing to quit. Significance is therefore high. This application has many significant strengths, including its focus on broad implementability, basis in a strong pilot (R34), encouragingly strong pilot outcomes, thoughtful adjustments in response to those findings, careful basis in existing literature, solid and much-needed focus on gender differences in response to smoking cessation interventions, and sophisticated analysis of online social dynamics as exploratory aims. Weaknesses are present but are relatively minor, and include unclear rationale for some changes (for example, making the previously organic "buddy" process artificial and investigator-directed) when the pilot results were so strong; failure to provide data regarding the proportion of participants who are likely to provide cotinine data in the manner proposed; and a PI with a unique background who—although certainly not lacking in overall experience—has limited experience leading large-scale NIH studies. Although the PI did not include a senior colleague from her home institution on the proposed team, concerns in this regard are largely attenuated by Dr. Prochaska's involvement as an MPI at 30% time throughout the award period.

Protections for Human Subjects:

Acceptable Risks and/or Adequate Protections

- The minimal risks in this study are adequately acknowleged and addressed.

Data and Safety Monitoring Plan (Applicable for Clinical Trials Only):

Acceptable

- The investigators propose a DSM plan, under which MPIs Pechmann and Prochaska will be responsible for plan administration.

Inclusion of Women, Minorities and Children:

- Sex/Gender: Distribution justified scientifically
- Race/Ethnicity: Distribution justified scientifically
- Inclusion/Exclusion of Children under 21: Including ages < 21 justified scientifically

- The investigators propose targeting their internet ads such that approximately 35% of participants will be of minority status. By design, 63% of participants will be women. Children age 18-20 will be included.

Vertebrate Animals:

Not Applicable (No Vertebrate Animals)

Biohazards:

Not Applicable (No Biohazards)

Resource Sharing Plans:

Acceptable

- De-identified data will be made available to other researchers.

Budget and Period of Support:

Recommend as Requested

Additional Comments to Applicant (Optional):

- Please be sure to only include relevant information in the Protection of Human Subjects section, and avoid including general methodological information.

THE FOLLOWING SECTIONS WERE PREPARED BY THE SCIENTIFIC REVIEW OFFICER TO SUMMARIZE THE OUTCOME OF DISCUSSIONS OF THE REVIEW COMMITTEE, OR REVIEWERS' WRITTEN CRITIQUES, ON THE FOLLOWING ISSUES:

PROTECTION OF HUMAN SUBJECTS (Resume): ACCEPTABLE

INCLUSION OF WOMEN PLAN (Resume): ACCEPTABLE

INCLUSION OF MINORITIES PLAN (Resume): ACCEPTABLE

INCLUSION OF CHILDREN PLAN (Resume): ACCEPTABLE

COMMITTEE BUDGET RECOMMENDATIONS: The budget was recommended as requested.

ADMINISTRATIVE NOTE:
During the review of this application, reviewers noted that: 1) page limits for one or more sections of the application may have been circumvented by including excess text in one or more application sections that do not have specified page limits (e.g., Protection of Human Subjects), and/or 2) the appendix may have been used to circumvent the page limits of the Research Plan. In egregious cases, the NIH has the authority to withdraw such applications from review or consideration for funding (NOT-OD-11-021). See concerns of Reviewer 3.

NIH has modified its policy regarding the receipt of resubmissions (amended applications). See Guide Notice NOT-OD-14-074 at http://grants.nih.gov/grants/guide/notice-files/NOT-OD-14-074.html. The impact/priority score is calculated after discussion of an application by averaging the overall scores (1-9) given by all voting reviewers on the committee and multiplying by 10. The criterion scores are submitted prior to the meeting by the individual reviewers assigned to an application, and are not discussed specifically at the review meeting or calculated into the overall impact score. Some applications also receive a percentile ranking. For details on the review process, see http://grants.nih.gov/grants/peer_review_process.htm#scoring.

MEETING ROSTER

Interventions to Prevent and Treat Addictions Study Section
Risk, Prevention and Health Behavior Integrated Review Group
CENTER FOR SCIENTIFIC REVIEW
IPTA
October 08, 2015 - October 09, 2015

CHAIRPERSON

PATTEN, CHRISTI A, PHD
PROFESSOR
DEPARTMENT OF PSYCHIATRY AND PSYCHOLOGY
MAYO CLINIC
ROCHESTER, MN 55905

MEMBERS

BRADIZZA, CLARA M, PHD *
SENIOR RESEARCH SCIENTIST
RESEARCH INSTITUTE ON ADDICTIONS
UNIVERSITY AT BUFFALO
STATE UNIVERSITY OF NEW YORK
BUFFALO, NY 14203

CARROLL, KATHLEEN M, PHD
PROFESSOR
DEPARTMENT OF PSYCHIATRY
SCHOOL OF MEDICINE
YALE UNIVERSITY
WEST HAVEN, CT 06516

COLLINS, BRADLEY N, PHD *
ASSOCIATE PROFESSOR
HEALTH BEHAVIOR RESEARCH CLINIC
COLLEGE OF PUBLIC HEALTH
TEMPLE UNIVERSITY
PHILADELPHIA, PA 19122

COMPTON, MARGARET A, PHD
PROFESSOR AND ASSOCIATE DEAN
DEPARTMENT OF NURSING
SCHOOL OF NURSING AND HEALTH STUDIES
GEORGETOWN UNIVERSITY
WASHINGTON, DC 20057

DONOHUE, BRADLEY C, PHD *
PROFESSOR
DEPARTMENT OF PSYCHOLOGY
UNIVERSITY OF NEVADA, LAS VEGAS
LAS VEGAS, NV 89117

FU, STEVEN , MD *
ASSOCIATE PROFESSOR
DEPARTMENT OF MEDICINE
UNIVERSITY OF MINNESOTA MEDICAL SCHOOL
MINNEAPOLIS, MN 55417

GRAY, KEVIN M, MD
ASSOCIATE PROFESSOR
DEPARTMENT OF PSYCHIATRY AND BEHAVIORAL SCIENCES
MEDICAL UNIVERSITY OF SOUTH CAROLINA
CHARLESTON, SC 29425

HEIL, SARAH H, PHD
ASSOCIATE PROFESSOR
DEPARTMENT OF PSYCHIATRY
COLLEGE OF MEDICINE
UNIVERSITY OF VERMONT
BURLINGTON, VT 05401

HETTEMA, JENNIFER E, PHD *
ASSOCIATE RESEARCH PROFESSOR
FAMILY AND COMMUNITY MEDICINE
UNIVERSITY OF NEW MEXICO
ALBUQUERQUE, NM 87109

JORENBY, DOUGLAS E, PHD *
PROFESSOR
DIRECTOR OF CLINICAL SERVICES
CENTER FOR TOBACCO RESEARCH AND INTERVENTION
SCHOOL OF MEDICINE AND PUBLIC HEALTH
UNIVERSITY OF WISCONSIN
MADISON, WI 53711

LEONARD, NOELLE R, PHD *
SENIOR RESEARCH SCIENTIST
COLLEGE OF NURSING
NEW YORK UNIVERSITY
NEW YORK, NY 10010

LEVIN, FRANCES R, MD
KENNEDY-LEAVY PROFESSOR
DEPARTMENT OF PSYCHIATRY
DIVISION OF SUBSTANCE ABUSE
COLLEGE OF PHYSICIANS AND SURGEONS
COLUMBIA UNIVERSITY NY STATE PSYCHIATRIC INSTITUTE
NEW YORK, NY 10032

MCKAY, JAMES R, PHD
PROFESSOR
DEPARTMENT OF PSYCHIATRY
UNIVERSITY OF PENNSYLVANIA
PHILADELPHIA, PA 19104

MCPHERSON, STERLING M, PHD *
ASSISTANT PROFESSOR
PROGRAM OF EXCELLENCE
IN ADDICTIONS RESEARCH
WASHINGTON STATE UNIVERSITY
SPOKANE, WA 99210

MENDELSON, TAMAR , PHD *
ASSOCIATE PROFESSOR
DEPARTMENT OF MENTAL HEALTH
JOHNS HOPKINS BLOOMBERG SCHOOL OF PUBLIC HEALTH
BALTIMORE, MD 21205

MURAMOTO, MYRA L, MD *
PROFESSOR
DEPARTMENT OF FAMILY
AND COMMUNITY MEDICINE
COLLEGE OF MEDICINE
UNIVERSITY OF ARIZONA
TUCSON, AZ 85719

OKUYEMI, KOLAWOLE S, MPH
PROFESSOR
DEPARTMENT OF FAMILY MEDICINE
AND COMMUNITY HEALTH
UNIVERSITY OF MINNESOTA
MINNEAPOLIS, MN 55414

ONDERSMA, STEVEN J, PHD *
PROFESSOR
MERRILL PALMER SKILLMAN INSTITUTE
SCHOOL OF MEDICINE
WAYNE STATE UNIVERSITY
DETROIT, MI 48236

POTTER, JENNIFER S, PHD *
ASSOCIATE PROFESSOR
DEPARTMENT OF PSYCHIATRY
HEALTH SCIENCE CENTER AT SAN ANTONIO
UNIVERSITY OF TEXAS
SAN ANTONIO, TX 78229

SHOPTAW, STEVEN , PHD
PROFESSOR
DEPARTMENT OF FAMILY MEDICINE
UNIVERSITY OF CALIFORNIA, LOS ANGELES
LOS ANGELES, CA 90024

STOOPS, WILLIAM W, PHD
ASSOCIATE PROFESSOR
DEPARTMENT OF BEHAVIORAL SCIENCE
UNIVERSITY OF KENTUCKY
LEXINGTON, KY 40536

STOTTS, ANGELA L, PHD
PROFESSOR
DEPARTMENT OF FAMILY AND COMMUNITY MEDICINE
UNIVERSITY OF TEXAS
HOUSTON, TX 77030

STRONG, DAVID R, PHD *
ASSOCIATE PROFESSOR
DEPARTMENT OF FAMILY AND PREVENTIVE MEDICINE
UNIVERSITY OF CALIFORNIA, SAN DIEGO
SAN DIEGO, CA 92093

TAXMAN, FAYE S, PHD
UNIVERSITY PROFESSOR
DEPARTMENT OF CRIMINOLOGY
LAW AND SOCIETY
GEORGE MASON UNIVERSITY
MANASSAS, VA 20110

TINDLE, HILARY A, MPH, MD *
ASSOCIATE PROFESSOR
DEPARTMENT OF MEDICINE
VANDERBILT UNIVERSITY
NASHVILLE, TN 37203

TURRISI, ROBERT J, PHD
PROFESSOR
DEPARTMENT OF BEHAVIORAL HEALTH
AND PREVENTION RESEARCH
PENNSYLVANIA STATE UNIVERSITY
UNIVERSITY PARK, PA 16802

VIDRINE, DAMON J, DRPH
ASSOCIATE PROFESSOR
DEPARTMENT OF FAMILY AND PREVENTIVE MEDICINE
UNIVERSITY OF OKLAHOMA HEALTH SCIENCES CENTER
OKLAHOMA CITY, OK 73104

WALDRON, HOLLY B, PHD
SENIOR SCIENTIST
OREGON RESEARCH INSTITUTE
EUGENE, OR 97403

WALTON, MAUREEN A, MPH, PHD
ASSOCIATE PROFESSOR
DEPARTMENT OF PSYCHIATRY
UNIVERSITY OF MICHIGAN
ANN ARBOR, MI 48105

WEWERS, MARY E, PHD *
PROFESSOR
COLLEGE OF PUBLIC HEALTH
INSTITUTE FOR POPULATION RESEARCH
THE OHIO STATE UNIVERSITY
COLUMBUS, OH 43210

WINSTANLEY, ERIN L, PHD *
ASSISTANT PROFESSOR
JAMES WINKLE COLLEGE OF PHARMACY
UNIVERSITY OF CINCINNATI
CINCINNATI, OH 45267

YURGELUN-TODD, DEBORAH A, PHD
PROFESSOR
DEPARTMENT OF PSYCHIATRY
UNIVERSITY OF UTAH
SCHOOL OF MEDICINE
SALT LAKE CITY , UT 84108

MAIL REVIEWER(S)

ARNEDT, J. TODD , PHD
ASSOCIATE PROFESSOR
DEPARTMENTS OF PSYCHIATRY AND NEUROLOGY
UNIVERSITY OF MICHIGAN
ANN ARBOR, MI 48105

MASON, MICHAEL J, PHD
ASSOCIATE PROFESSOR & DIRECTOR
DEPARTMENT OF PSYCHIATRY
VIRGINIA COMMONWEALTH UNIVERSITY
RICHMOND, VA 23298

SCIENTIFIC REVIEW OFFICER

MINTZER, MIRIAM , PHD
SCIENTIFIC REVIEW OFFICER
CENTER FOR SCIENTIFIC REVIEW
NATIONAL INSTITUTES OF HEALTH
BETHESDA, MD 20892

NI, WEIJIA , PHD
CHIEF/SCIENTIFIC REVIEW OFFICER
RISK, PREVENTION AND HEALTH BEHAVIOR
INTERGRATED REVIEW GROUP
CENTER FOR SCIENTIFIC REVIEW
NATIONAL INSTITUTES OF HEALTH
BETHESDA, MD 20892

EXTRAMURAL SUPPORT ASSISTANT

WATTS, MELISSA D
EXTRAMURAL SUPPORT ASSISTANT
CENTER FOR SCIENTIFIC REVIEW
NATIONAL INSTITUTE FOR HEALTH
BETHESDA, MD 20892

* Temporary Member. For grant applications, temporary members may participate in the entire meeting or may review only selected applications as needed.

Consultants are required to absent themselves from the room during the review of any application if their presence would constitute or appear to constitute a conflict of interest.
